# Maillard reaction between oligopeptides and reducing sugar at body temperature: The putative anti-glycation agents

**DOI:** 10.3389/fnut.2022.1062777

**Published:** 2022-12-01

**Authors:** Qiang Wang, Xiaofeng Xiang, Yuejie Xie, Kai Wang, Cao Wang, Xuyuan Nie, Puzhi Wang

**Affiliations:** ^1^Key Laboratory of Lipid Resources Utilization and Children’s Daily Chemicals, Chongqing University of Education, Chongqing, China; ^2^College of Modern Health Industry, Chongqing University of Education, Chongqing, China; ^3^Chongqing Sanyi Food Co., Ltd., Chongqing, China

**Keywords:** diabetes, Maillard reaction, glucose, carnosine, bioactive peptide

## Abstract

Type 2 Diabetes mellitus (T2DM) is one of the most common chronic multifactorial diseases, which is associated with the increased concentration of glucose in the blood. Therefore, the utilization of blood lowering agents is clearly a promising approach which can lead to a suppression of the evaluated blood glucose, and thus curing T2DM and other complication. In this study, we evaluated the glucose lowering effect of a varieties of amino acids (alanine and histidine), dipeptides (carnosine and α-alanine-L-histidine), and tripeptide (glutathione) by reacting with glucose, fructose, and sucrose under 37°C and pH 7.4 to mimic their reaction in physiological condition. By measuring the reduction of reactants and the formation of Maillard reaction products over the course of 21 days’ storage, we found that the glucose lowering effect of carnosine was better than the counterparts. The histidine residue in carnosine may contribute to its glucose lowing effect while β-amino acid β-alanine residue could facilitate the glucose lowering effect of carnosine by maintaining its chemical stability during the storage. These results may open up new avenues for the applications of bioactive peptide carnosine as a natural blood sugar lowering agent to control T2DM.

## Introduction

Diabetes mellitus (DM) is one of the most challenging chronic multifactorial diseases, which becomes a global threat to public health in the 21st century (1). According to the International Diabetes Federation (IDF) estimation, about 536.6 million people worldwide suffered from DM in 2021, which has estimated to be 783.2 million in 2045 (2). Among them, type 2 diabetes mellitus (T2DM) is the most prevalent subtype which accounts for approximately 90% of DM (2). It is generally described as a human hyperglycaemic state characterized by insulin resistance in peripheral tissues. Besides, it is strongly influenced by the complex interplay of genetics of individuals and environmental factors. The increased concentration of glucose in human blood is one of the most significant characteristic features of T2DM. The elevated glucose levels may promote the formation of advanced glycation end products (AGEs), a group of stable cross-linked products primarily formed by the reaction between reducing sugars (e.g., glucose) and proteins. AGEs are a potential cause of the maladies the formation of which is associated with the high blood sugar content resulting from diabetes. Therefore, how to effectively lower the blood sugar is clearly a promising approach to cure T2DM and other complications (3).

Whereas first- and second-line glucose-lowering drugs, such as metformin and pioglitazone have been commonly prescribed to treat T2DM in clinical practice, their adverse effects have emerged as a major concern for public health (4). Therefore, numerous researches have been set forth to explore novel natural agents that can effectively reduce blood glucose but with less side effects (5). For instance, some phytochemicals, such as berberine, curcumin, catechin, polyphenols, etc., have displayed certain levels of beneficial effects for the management of T2DM (6, 7). Oligopeptides have been suggested to carry a wide range of functional and biological properties, such as antimicrobial, antioxidative, antithrombotic, antihypertensive and immunomodulatory activities, etc (8, 9). However, the glucose lowering effect of oligopeptides has yet to be investigated. Previous study implied that dipeptide carnosine was able to readily react with reducing sugars, including glucose, galactose, deoxyribose, and triose dihydroxyacetones, and thus could be as a potential anti-protein cross-linking agent. Such investigation, however, was carried out under an unphysiological temperatures (60°C) and sugar concentrations (20 mM) (10).

In order to understand if oligopeptides could react with blood sugar to inhibit its reaction with proteins for the formation of AGEs, a more realistic condition should be employed. In this work, we studied the reactions between oligopeptides and various sugars including reducing glucose and fructose, as well as non-reducing sucrose under 37°C and pH 7.4 to imitate their processing in physiological condition. In addition, we examined the reactivity of carnosine with different concentrations of glucose to mimic the normal and diabetic blood sugar levels. For comparison, we also studied the reactions of amino acids, dipeptides, and tripeptides with glucose. The findings could shed light on the utilization of bioactive oligopeptides as glucose-lowering agents.

## Materials and methods

### Chemicals

Alpha-alanyl-histidine (Ala-His, G-1245) was purchased from Bachem Americas, Inc., (Torrance, CA). 4-Fluoro-7-sulfamoylbenzofurazan (ABD-F) was purchased from Wako Chemicals USA, Inc., (Richmond, VA, USA). L-carnosine (>99%), L-alanine (>98%), L-histidine (>99%), L-glutathione reduced (GSH), glucose, fructose, sucrose, o-phthalaldehyde (OPA, solution complete), 3,5-dinitrosalicylic acid (DNS), diethylenetriaminepentaacetic acid (DTPA), sodium hydroxide, sodium potassium tartrate, sodium potassium tartrate, sodium phosphate dibasic (Na_2_HPO_4_), sodium chloride (NaCl), potassium chloride (KCl), potassium phosphate monobasic (KH_2_PO_4_), and sodium azide were obtained from Sigma-Aldrich Co., (St. Louis, MO, USA). All solutions were prepared using ultrapure distilled deionized water (18.2 MΩ cm).

### Preparation of reaction solutions

Carnosine, α-alanyl-histidine, L-alanine, L-histidine, and glutathione (20 mM) in phosphate buffer (137 mM NaCl, 2.7 mM KCl, 4.3 mM Na_2_HPO_4_, and 1.4 mM KH_2_PO_4_, pH 7.4) were mixed with equal volume of sugar (glucose, fructose, or sucrose) at various concentrations (0, 5, 15, and 30 mM) in the same phosphate buffer solution. Sodium azide (0.02 wt%) was added into the mixture to inhibit microbial growth. The mixed solution (10 mL) was transferred to glass test tube, which was then capped and stored in a walk-in incubator room at 37°C. After the designed storage period, the tubes were immediately kept at −20°C freezer for further analysis. All model systems were prepared in triplicate.

### Determination of amino acids, dipeptides, and glutathione in solutions

The analysis of amino acids, dipeptides, and GSH was based on their derivatization with o-phthaldialdehyde (OPA) to form a high fluorescent adduct, following the method of Kuerban et al. (11). In brief, derivatizations of blank and sample solutions were performed by mixing 0.2 mL sample solution with 1.5 mL OPA solution, which was then stirred thoroughly. After the fixed 2 min reaction time at a room temperature (22°C), the fluorescence intensity of OPA derivative solution was then recorded at an excitation wavelength of 348 nm and at an emission wavelengths of 450 nm using PTI QM-3 luminescence spectrofluorometer (Photon Technology International, NJ, USA). Spectral bandwidths of 1.0 and 1.0 nm were set for the excitation and the emission slits, respectively. The integration time in both was 1 s. The wavelength increment was 1 nm when running the spectrum scanning. The intensity of the spectra was determined as the emission signal intensity (counts per second) measured by means of a photomultiplier, and the average intensity of 15 s was reported. All measurements were performed in duplicate.

### Determination of sugar content in solutions

Sugars concentrations in each system during the storage were measured using a dinitrosalicylic acid (DNS) colorimetric method (12). Briefly, sodium potassium tartrate solution was prepared by dissolving 135 g of sodium potassium tartrate in 225 mL of H_2_O. DNS solution was prepared by dissolving 4.5 g of DNS reagent in 90 mL of 2 M NaOH. DNS reagent was prepared by mixing the sodium potassium tartrate solution and DNS solution to make up the volume to 450 mL with water, which was then stored in dark before using. To determine the concentration of sugar, 1.5 mL sample solution was first mixed with 3 mL DNS reagent in test tube. After heated for 5 min at 100°C, the mixture was cooled down to a room temperature. Finally, 0.2 mL mixture was withdrawn and diluted with 2.5 mL distilled water. The absorbance of the diluted solution and standards (glucose or fructose) was measured at 540 nm against blank using a UV-Vis scanning spectrophotometer (Shimadzu UV-2101PC UV-VIS, Kyoto, Japan).

### Determination of Maillard reaction products

Fluorescence of MRPs in sample solution was determined using PTI QM-3 luminescence spectrofluorometer (Photon Technology International, NJ, USA). The set for the PTI fluorimeter was the same as the above mentioned methods except that the excitation and emission wavelength were set as 386 and 447 nm, respectively (13). For the blank, the assay was conducted in the same manner, but milli-Q water was added instead of MRPs.

### Determination of cysteine residue content in glutathione

Cysteine residues in glutathione (GSH) were measured directly using a sensitive fluorescent probe 4-fluoro-7-sulfamoylbenzofurazan (14). In short, sample solution was derivatized with ABD-F and measured directly at an excitation wavelength of 365 nm and at emission wavelengths of 492 nm by PTI QM-3 luminescence spectrofluorometer (Photon Technology International, NJ, USA) following dilution by phosphate buffer (100 mM phosphate buffer, pH 8.0, and 1 mM diethylenetriaminepentaacetic acid). The ABD-F thiol probe was added (10 μL of 10 mM ABD-F in PBS) to the sample solutions, mixed by vortex, and incubated in a 60°C water bath for 20 min. The parameter setup for the PTI fluorimeter was the same as the above mentioned methods.

### Measurement of browning products

To follow the color formation during the storage, the visible absorption at 420 nm were determined in the reaction mixtures using UV-VIS scanning spectrophotometer (Shimadzu UV-2101PC UV-VIS, Kyoto, Japan) (15).

### Statistical analysis

All the data obtained were expressed as mean ± SD. Statistical analysis was performed by one-way analysis of variance (ANOVA) followed by Duncan’s multiple range test using IBM SPSS statistics 23 for Windows (SPSS, Chicago, IL, USA). *P*-value at a less than 0.05 was considered to be statistically significant.

## Results and discussion

### The effect of glucose concentration on Maillard reaction with carnosine

**FIGURE 1:**
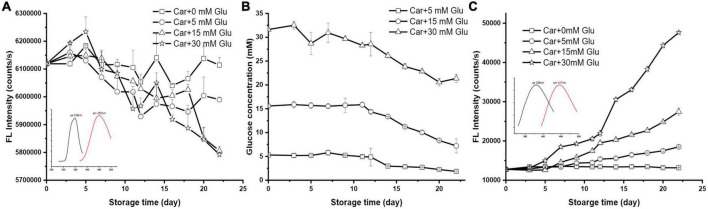
The changes of **(A)** carnosine florescence intensity, **(B)** glucose concentration, and **(C)** the formation of MRP during storage (the initial mixture contained 10 mM carnosine with 0, 5, 15, or 30 mM of glucose; inserted EX/EM scan was OPA derivatized carnosine and MRPs).

The reaction between carnosine and glucose with various concentrations (i.e., 0, 5, 15, and 30 mM) in PBS buffer (pH 7.4) was carried out at 37°C. These three levels of glucose represent normal, diabetic, and abnormal blood glucose level, respectively. The depletion of carnosine and glucose, and the formation of Maillard reaction products (MRPs) were determined by fluorimeter and spectrometer as shown in Figure 1.

In the absence of glucose, the concentration of carnosine remained steady during the storage (Figure 1A). The mixture of 10 mM carnosine with glucose at 37°C and pH 7.4 could undergo reactions resulting in a significant reduction in both carnosine and glucose concentrations. For instance, for the system with combination of 10 mM carnosine and 5 mM glucose, carnosine and glucose depleted 2.11 and 64.96%, respectively, after 22 days’ storage (Figures 1A,B). The addition of 15 mM glucose slightly enhanced the depletion of carnosine to 5.11%, but significantly decreased the depletion of glucose to 53.77%. Further increasing the concentration of glucose to 30 mM caused an enhancement of carnosine depletion to a degree (i.e., 5.33%) that was not different from that of lesser glucose concentration. However, the depletion of glucose was drastically retarded as judged by the 32.49% of glucose lost in such system.

On the other hand, the generation of fluorescent compounds in samples during the storage time were identified through fluorescence excitation spectra, and a characteristic maxima excitation wavelength of 386 nm was recorded (Figure 1C inserted black line). Following excitation wavelength at 386 nm, the samples exhibited emission wavelength of 447 nm (Figure 1C inserted red line). However, carnosine alone did not generate any fluorescence under the same reaction conditions. This results indicate the development of Maillard reaction products (MPRs) through the glycation of carnosine with glucose. Therefore, the fluorescence intensity of MPRs in the samples were monitored at 386 nm excitation and 447 nm emission during storage. According to Figure 1C, a remarkable increase in MRPs fluorescence intensity was observed in a dose dependent manner. In particular, the incubation of 10 mM carnosine with normal concentration (i.e., 5 mM) of glucose resulted in a gradual and steady increment in the fluorescence intensity. An increase of 44.99% fluorescence intensity was observed after 22 days’ storage when compared to carnosine alone (Figure 1C). When raising the glucose concentration to diabetic (15 mM) and abnormal (30 mM) blood sugar levels, the fluorescence intensity increased 115.03 and 272.97%, respectively, after 22 days’ storage. A significant change in fluorescence intensity was observed in all the conditions after 12 days’ storage.

Generally, the occurrence of Maillard reaction between reducing sugar and amine starts with the formation of Schiff base, followed by the Amadori rearrangement of the highly unstable Schiff base into early intermediate glycation products known as Amadori product (16). As intermediate products, Amadori products undergo degradation over times which generate covalent cross-linked and fluorescent derivatives known as MPRs (17). Currently, there is in lack of both universally accepted method to detect MPRs and internal standards or internationally recognized standard unit of measurement. Consequently, the detection of fluorescent product is a popular method for Maillard reaction. It is widely accepted that pentosidine is an MPRs marker, which is excited at 370 nm and emitted at 440 nm (18, 19). The fluorescence results from this study are similar to that of pentosidine which confirmed the formation of MPRs products.

The browning colorants generally associated with the occurrence of advanced stage Maillard reaction, such as advanced glycation end products (AGEs) was not observed. Alternatively, the color of all the mixtures as well as the controls remained clear during the storage. This was also confirmed by measuring the absorbance of the mixtures at 420 nm during storage. Numerous studies on Maillard reaction suggest that the development of fluorescent compounds occurs in the Maillard reaction prior to the generation of brown pigments (20). Besides, fluorescence of MRPs will disappear after reaching a maximum with increased heating time (21). The disappearance of fluorescence will be accompanied by the formation of browning products. In this study, the constant increasing in florescent intensity during incubation time coincides with the absence of browning pigments, suggesting the formation of unfavorable polymers by AGEs will not occur in such a physiological condition.

All these results suggest that chemical reaction could occur between carnosine and reducing sugar, presumably through sugar-amine Maillard reactions at body temperature over a long period of storage. In addition, the effect of carnosine to lower glucose could be observed both upon normal and diabetic blood sugar levels. However, this effect could be antagonized under extremely high glucose concentration.

### The effect of sugar type on Maillard reaction with carnosine

**FIGURE 2:**
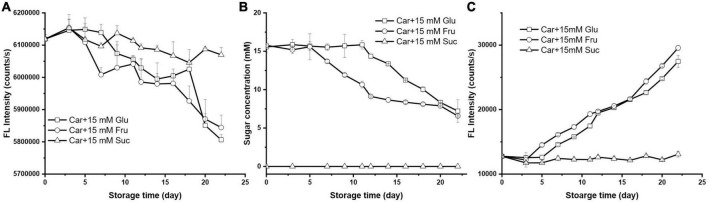
The changes of **(A)** carnosine florescence intensity, **(B)** sugar concentration, and **(C)** the formation of MRP during storage (the initial mixture contained 10 mM carnosine with 15 mM glucose, fructose, or sucrose).

To gain insight in the role of sugar type on the Maillard reaction with carnosine, hexose glucose and its ketose isomer fructose as well as disaccharide sucrose were selected for comparison. The results of the interaction between 10 mM carnosine and these three sugar at a fixed concentration of 15 mM at 37°C were presented in Figure 2.

The concentration of carnosine in the system was characterized by the fluorescence intensity loss of OPA derivatized carnosine (Figure 2A). As can be seen, the concentration of carnosine did not change significantly within 7 days of storage when 15 mM sucrose was present. Meanwhile, no reducing sugar was detected during the storage suggesting the entity of sucrose (Figure 2B). The absence of fluorescence products in the reactants also indicated that there was no chemical reaction between carnosine and sucrose (Figure 2C). In fact, this is not surprising since Maillard reaction only happens between reducing sugar and α/ε-amine (22).

When it came to fructose, 4.48% of carnosine was consumed by the end of the storage (Figure 2A), which was similar to that of its isomer glucose. However, the ability of fructose to consume carnosine was stronger than that of glucose before 12 days’ storage after which they had similar impact (Figure 2B). At day 12, the fructose caused 3.15% decline in carnosine, whereas only 1.46% was observed in the presence of glucose, manifesting the faster reaction rate of the former. The concentration of fructose decreased dramatically after 5 days’ storage and leveled off after day 12. Conversely, the lag phase (11 days) for glucose was much longer than that of fructose (5 days), and the concentration of glucose was constant in the beginning and started to drop at day 11. Both sugar shared similar concentrations in the system after 20 days’ storage. The formation of MPRs in carnosine/fructose system was characterized by strong fluorescence intensity at excitation 386 nm and emission 447 nm which was associated with pentosidine compounds. The intensity of carnosine with fructose was stronger than that with glucose (Figure 2C). All these results suggested that fructose reacted faster than glucose with carnosine. Previous study reported the prevailing propensity of pentose over hexose when participating in Maillard reaction. For instance, Namli et al. (23) reported that the reactivity of fructose was faster than glucose for glycation with soy protein isolate. Earlier study from Benjakul et al. also found fructose was more reactive than fructose in forming the fluorescent intermediate compounds, and they postulated that it was due to the higher proportion of open chain form in fructose (20). In consequence, an amino acid–sugar complex could be formed more easily with fructose rather than glucose.

### The role of amino acids on Maillard reaction with glucose

Besides the saccharide substances, the composition of peptide will also influence the overall reactivity. To elucidate if the role of carnosine on glucose lowering is related to its β-alanine-L-histidine composition, the reaction of 15 mM glucose with 10 mM of each individual amino acid of carnosine (i.e., alanine and histidine) was studied.

**FIGURE 3:**
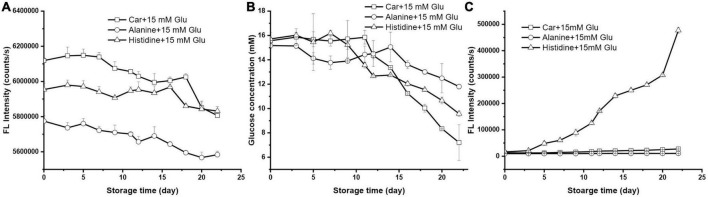
The changes of **(A)** amino acids florescence intensity, **(B)** glucose concentration, and **(C)** the formation of MRP during storage (the initial mixture contained 10 mM carnosine, alanine, or histidine with 15 mM glucose).

As illustrated in Figure 3A, OPA derivatized amino acid displayed variable fluorescence intensity. After 22 days’ storage, histidine and alanine decreased 2.05 and 3.31%, respectively, both of which had a lower rate than carnosine. Among three systems, alanine had the weakest effect to lower the glucose level, followed by histidine, being 22.24 and 39.09%, respectively (Figure 3B). Figure 3C showed that histidine drastically evoked the generation of fluorescence intensity corresponding to the formation of unfavorable AGEs; while the counterpart had negligible contribution. All these results together implied that histidine had a greater tendency to interact with glucose than did alanine; while dipeptide carnosine had the greatest reactivity among the three to react with glucose to only form early stage MPRs.

The effect of amino acid type on the Maillard reaction was investigated previously. For instance, Kwak et al. determined the Maillard reaction of 5 sugars with 12 amino acids and found that the reactivity of lysine was 2–3 times higher than others because of its two amino groups (α- and ε-amino groups) (24). However, very few of study compared the reactivity of amino acid and dipeptide, particularly histidine containing dipeptide in Maillard Reaction. One study compared the reactivity of 8 lysine-containing dipeptides (Lys-x) and the individual amino acid to react with various sugars (25). Their results showed that not only the reaction rate but also the degree of Maillard reaction were greater for all the dipeptides than for the corresponding amino acid, which coincided with our results. In general, Maillard reaction begins with the reaction of a primary amino group with a sugar carbonyl to form a Schiff base. This unstable intermediates will rearrange into the stable Amadori or Heyn’s products (26). Previous study stated that the early stage of the Maillard reaction between dipeptides and reducing sugar was affected by the acidity of the second amino acid residue (27). For a dipeptide, the initial imine in a positively charged basic amino acid in second position was converted to the ketoamine to a higher extent. Conversely, the presence of a negative or neutral charge at the second residue slowed down the Maillard reaction. In our case, it is reasonable that the basic histidine had a higher reaction rate with glucose when compared with its neutral counterpart alanine. This is because of the proximity of a protonated basic group resulted in an enhanced Amadori rearrangement.

### The role of carnosine isomer and glutathione on Maillard reaction with glucose

Earlier study proposed that the bioactivity of carnosine is due to the unique β-alanine-L-histidine structures (28). In order to verify if this is also the case in blood glucose lowering effect, we compared the glucose lowering effect of carnosine with its isomer α-alanine-L-histidine and a tripeptide reduced glutathione (GSH).

**FIGURE 4:**
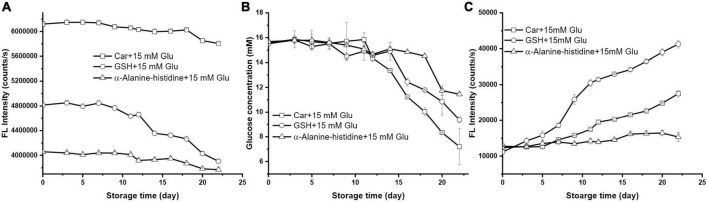
The changes of **(A)** GSH florescence intensity, **(B)** glucose concentration, and **(C)** the formation of MRP during storage (the initial mixture contained 10 mM carnosine, GSH, alpha-alanine-histidine with 15 mM glucose).

As shown in Figure 4A, both α-alanine-L-histidine and GSH had greater loss rate than carnosine upon reacting with glucose. After 22 days’ storage, the amount of GSH and α-alanine-L-histidine, as indicated by the intensity loss of OPA derivatized fluorescence products, plummeted 18.93 and 7.12%, respectively. However, carnosine induced the greatest loss of glucose, followed by GSH; while its isomer had the weakest potency to reduce glucose (Figure 4B). Similar as carnosine, the depletion of α-alanine-L-histidine and GSH when encountering with glucose was due to the Maillard reaction which can be supported by the formation of fluorescence products (Figure 4C). According to Figure 4C, GSH had the highest propensity to form MRPs among the three. Previous study showed that the released hydrogen sulfide from the cysteine residue in GSH was a key precursor for Strecker degradation, thus accelerating the generation of MRPs (29). Nevertheless, its florescence intensity was 10-fold less than that formed by histidine after same storage time (Figure 3C). To the best of our knowledge, the study on the Maillard reaction between reducing sugar and α- or β-amino acid is scarce to none. But some study found that D-amino acids are formed on heating aqueous solutions of L-amino acids with a variety of saccharides such as glucose, fructose, and saccharose at 100°C (30). Such phenomenon also explains the formation of D-amino acids which is the result of Maillard reaction. The conversion of D-amino acids to L-amino acids which is commonly referred to as racemization, requires chiral carbo center (31). It is anticipated that the occurrence of racemization could potentially retard the Maillard reaction rate of amino acids with reducing sugar. In this study, the β-alanine on carnosine molecule has no stereocenter, meaning it cannot go through racemization during Maillard reaction, which presumably be the reason for its greater glucose lowering effect than its counterpart α-alanine-L-histidine.

Meanwhile, previous study suggested that glutathione, a tripeptide containing cysteine, is a potential bioactive peptide to modulate the Maillard reaction (16). For instance, a recent study found that glutathione could trap α-dicarbonyl deoxypentosones to form stable glutathione-deoxypentosone adduct, thus inhibiting the formation of AGEs (32). The cysteine residue, particularly the free sulfhydryl groups of cysteine are the key site of glutathione to react with deoxypentosones. Therefore, we measured the amount of cysteine in the absence and presence of glucose during storage time. As shown in Table 1, the content of cysteine in both systems at the same storage time was quite similar which was independent of glucose, which suggested that the role of GSH to react with glucose was not because of the cysteine residues. We also noticed that the amount of cysteine in both systems declined as the extend of storage time. This is not surprising since the reduced thiols are easily oxidized to disulfides. The results from Kamińska et al. indicated that the total GSH concentration quickly decreased particularly in the presence of catalytic agents at room temperature (33). Our results suggest that GSH may not be an ideal glucose lower agent in the physiological conditions although it may mitigate the formation of AGEs in a higher temperature.

**TABLE 1:** The change of thiols on GSH in the absence and presence of 15 mM glucose.

Storage time (day)	GSH	GSH + 15 mM Glu
0	913385.7 ± 7940.1^a^	929536.7 ± 12384.4^a^
3	882340.3 ± 2151.7^a^	917367.7 ± 10276.6^a^
5	867521.5 ± 4843.5^a^	891137.0 ± 1916.5^a^
7	848418.0 ± 364.3^a^	867279.3 ± 3201.0^a^
9	842086.7 ± 3677.3^a^	841746.7 ± 12109.7^a^
11	840908.7 ± 310.0^a^	844793.5 ± 4917.5^a^
12	849437.3 ± 13.5^a^	822762.3 ± 4640.7^a^
14	842978.7 ± 51.0^a^	809599.7 ± 1995.1^b^
16	837948.5 ± 198.5^a^	797215 ± 2147.6^b^
18	810932.3 ± 5171.8^a^	797272.5 ± 4676.5^a^
20	810132.3 ± 2502.8^a^	780112.7 ± 2546.9^b^
22	786097.7 ± 4234.0^a^	771359 ± 8393.7^a^

The values with different superscript letters within a row are significantly different (*p* < 0.05).

## Conclusion

In this study, we systematically compared the glucose lowering effect of carnosine with different concentration of glucose (5, 15, and 30 mM) as well as with hexose/ketose isomers fructose and disaccharides. Our results found that carnosine only reacts with reducing sugar such as glucose and fructose. It is able to gradually decrease the glucose level at body temperature over the course of 21 days’ storage. Its reaction relies on the concentration of glucose, and the greatest effect occurs with glucose at a diabetic level (15 mM), which consumed 53.77% of glucose at the end of storage. To elucidate the underlying mechanism, we compared the glucose lowering effect of carnosine with individual amino acid (alanine and histidine), its counterpart (α-alanine-L-histidine), and tripeptide (glutathione). Our findings suggest that histidine but alanine in carnosine may contribute to its glucose lowing effect. However, β-alanine, because of its beta-amino acid nature could stabilize the chemical stability of carnosine during the storage, thus facilitating the glucose lowering effect of carnosine. This is in contrast to GSH, which degrades gradually through the oxidation of thiol group. Since no advance glycation end products were formed during storage, carnosine only forms early stage Maillard products with glucose. The findings from the current study indicate that carnosine is a potential bioactive peptide to control the diabetic blood sugar level without generating adverse effect.

## Data availability statement

The original contributions presented in the study are included in the article/supplementary material, further inquiries can be directed to the corresponding author.

## Author contributions

QW: conceptualization, writing, supervision, and funding acquisition. XX: investigation and writing. YX: methodology. KW and CW: data curation. XN and PW: funding acquisition. All authors contributed to the article and approved the submitted version.
